# Employing in-context learning prompts with large language models for drone routing in delivery services

**DOI:** 10.1371/journal.pone.0321917

**Published:** 2026-03-27

**Authors:** Mahmoud Masoud, Mohammed Elhenawy, Ahmed Abdelhay

**Affiliations:** 1 Department of Information Systems & Operations Management, King Fahd University of Petroleum & Minerals, Dhahran, Saudi Arabia; 2 Center for Smart Mobility and Logistics, King Fahd University of Petroleum & Minerals, Dhahran, Saudi Arabia; 3 Centre for Accident Research and Road Safety–Queensland, Queensland University of Technology, Kelvin Grove, Queensland, Australia; 4 Department of Computer Engineering, Minia University, Minia, Egypt; National Taiwan University of Science and Technology, TAIWAN

## Abstract

Autonomous Aerial Vehicles (AAVs) – known as drones – employment in delivery services is one of the promising transformative technologies. The AAV industry has taken significant steps to develop drones to fulfill the needs of delivery services. However, AAVs have limitations related to the flight range and payload capacity. Therefore, drone route planning is crucial to reducing the effectiveness of these challenges. The recent emergence of Large Language Models (LLMs) has opened new possibilities for solving combinatorial problems using in-context learning (ICL). Unlike traditional machine learning models, LLMs can generate solutions without requiring task-specific fine-tuning by leveraging solved examples within their input prompts. In this study, we explore the application of LLMs to the Drone Routing Problem (DRP), leveraging various ICL strategies to generate optimized delivery routes. Our solution ensures that drone routes are planned to reduce the traveling distance for the full route. Notably, it ensures that drones don’t mess any delivery points and fast delivery routes. Through extensive experimentation, we evaluate the effectiveness of different prompt engineering techniques in guiding LLMs to produce high-quality, non-hallucinated route plans. We compared our model results to heuristic-based generated routes to demonstrate the variation between our technique and other techniques. The results demonstrate that LLMs, when properly prompted, can reliably generate valid routing solutions, highlighting their potential as a flexible and adaptive tool for drone logistics planning. Project link: https://github.com/ahmed-abdulhuy/Solve-TSP-using-GPT3.5.git

## Introduction

The rapid advancement of drone technology has enabled a wide range of applications, including package delivery, surveillance, disaster response, and environmental monitoring [1–3]. One of the key challenges in these applications is the Drone Routing Problem (DRP), which involves determining optimal routes for drones to complete their tasks efficiently. Traditional approaches to solving DRP rely on heuristic algorithms [4], optimization-based methods [5,6], or reinforcement learning [7,8], often requiring extensive computational resources and domain-specific expertise.

While these methods have yielded valuable insights, they often encounter limitations in scalability and adaptability when applied to real-world scenarios. In contrast, recent breakthroughs in Large Language Models (LLMs), particularly through in-context learning (ICL), offer a promising alternative. LLMs can interpret and generate responses based on contextual examples, enabling them to tackle complex combinatorial optimization problems without the need for extensive task-specific fine-tuning.

LLM advancement introduced new possibilities for solving complex combinatorial optimization problems [9,10]. LLMs can offer flexible and data-efficient approaches to solving specific problem instances by leveraging in-context learning. Researchers tried a variety of methods to optimize LLMs’ responses to in-context prompts. Meanwhile, one group of response optimization methods depends on engineering in-context learning prompts fed to the model by using in-context learning techniques such as zero-shot, few-shot, chain of thoughts (CoT), etc. [11–17], other researchers investigate the potential of combining ensemble learning techniques with other in-context learning techniques to increase the optimality of the final response [15,18,19]. In several research papers, combining in-context learning techniques with LLM fine-tuning showed an increase in response accuracy [20–22].

This research investigates the application of in-context learning prompts within LLMs to solve the Drone Routing Problem. By leveraging prompt engineering strategies, we aim to guide LLMs in generating feasible and optimized routes for drone operations. Our study compares the performance of this LLM-based approach against conventional optimization methods, addressing key questions such as the feasibility of generating high-quality routes, the effectiveness of different prompt designs, and the overall potential of LLMs in enhancing drone routing efficiency.

Through comprehensive experimentation and analysis, our research seeks to bridge the gap between natural language processing and operational research, paving the way for innovative, AI-driven solutions to the complex challenges inherent in modern drone routing applications.

The main contribution of this paper is to investigate the potential of using LLMs such as GPT-3.5-turbo to plan optimal drone route plans as a travel salesman problem (TSP). Our contribution to this paper is as follows:

1. We created a dataset of simulated routes.
2. We worked on engineering in-context learning prompts with state-of-the-art prompting techniques.
3. We apply and validate self-ensemble techniques over a fine-tuned model.

**Fig 1:**
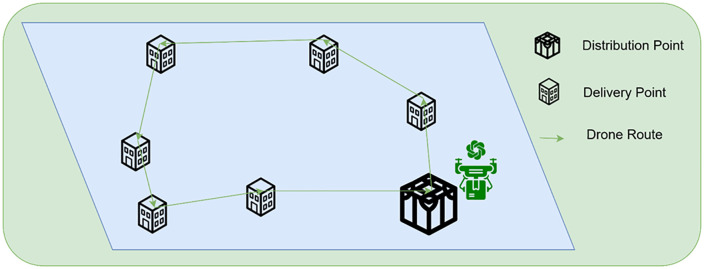
Representation of the Drone's route planning for a delivery system.

In addition to addressing the gap of limited studies on the use of LLMs for combinatorial problems, the following research questions drive this inquiry (Fig 1):

1. Can an LLM find a sub-optimal path based on its prior knowledge provided with different in-context learning techniques?
2. How does increasing station numbers affect LLM's effectiveness in delivering delivery routes?
3. Does self-ensemble enhance LLM's proposed routes?

## Literature review

The drone industry has experienced rapid growth in recent years, driven by advancements in autonomous navigation, battery efficiency, and artificial intelligence. One of the most transformative applications of drones is in last-mile delivery, where drones are deployed to transport goods directly to customers, reducing delivery times and costs [23]. However, drone delivery introduces unique challenges, including limited battery capacity, package weight constraints, and regulatory compliance. These factors necessitate the development of advanced drone routing algorithms that can optimize flight paths, balance energy consumption, and ensure timely and cost-effective deliveries [24].

The main technical issues for drone routing in drone-based delivery systems are coupled with trajectory design and battery capacity. The trajectory design considers efficient planning of the waypoint and visiting order of delivery stations according to the drone’s flight time [3].

One way to classify trajectory planning is based on delivery method type. Research has classified these methods as single drone-based delivery [25], truck drone collaborative delivery [26], public transport and drone collaborative delivery [8], and multi-drone-based delivery for multiple customers [27].

Overall route planning to visit delivery stations is derived from different variants of the traveling salesman problem (TSP) [26–28]. To address this problem, many mathematical models have been proposed to obtain a sub-optimal solution under different objectives, considering several constraints, such as the heuristic method [29,30], mixed integer linear Programming (MILP) [27], and reinforcement learning [8]. Due to the constrained battery payload and limited battery capacity, trajectory planning methodologies for drone-based delivery systems are adopted to minimize the average delivery time and maximize the coverage area that a drone can serve in a single charge [31].

Studies have demonstrated LLMs’ potential in solving routing problems, including the Traveling Salesman Problem (TSP) and Drone Routing Problem (VRP), by encoding problem constraints within textual prompts. Research has shown that LLMs can approximate near-optimal solutions when guided with structured prompt engineering. However, limited work has been conducted on applying LLMs to drone-specific routing problems, which involve additional complexities such as battery constraints, flight dynamics, and three-dimensional navigation [32].

Numerous researchers have explored the use of LLMs to solve combinatorial optimization problems. For instance, one study presented the first investigation of LLMs as evolution combinatorial optimizers by proposing an approach called LLM-driven Evolution Algorithm (LMEA) to solve the Traveling Salesman Problem (TSP) [33]. In this approach, the LLM selects parent solutions from the existing population and performs crossover and mutation operations to generate offspring solutions in each generation of the evolution search. The new solutions are then evaluated and incorporated into the population for subsequent generations. This method demonstrated competitive performance compared to traditional heuristics, achieving high-quality solutions on TSP instances with up to 20 nodes.

Another promising approach is PROmpting Optimization (OPRO), which leverages LLMs as optimizers by formulating optimization tasks in natural language [34]. In each optimization step, the LLM generates new solutions from a prompt containing previously generated solutions and their respective values. These new solutions are subsequently evaluated and appended to the prompt for the next iteration. This iterative approach allows the model to improve the quality of solutions over time.

While research on employing LLMs for combinatorial optimization is still in its early stages, most existing studies focus on generic optimization tasks. Our study is the first to investigate the potential of employing fine-tuned LLMs to address the Drone Routing Problem (DRP), specifically targeting drone delivery applications. By addressing this research gap, we contribute to the intersection of natural language processing (NLP) and optimization, providing insights into how LLMs can be utilized for solving real-world routing challenges in drone applications.

### Methodology

**Fig 2:**
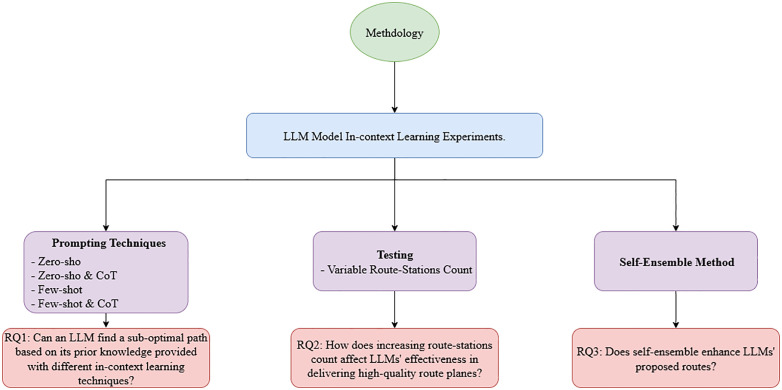
Methodology plan to answer research questions. Each experiment responds to one of the research questions.

As shown in Fig 2, our methodology, designed to address the research questions outlined in the introduction, utilizes the GPT-3.5-turbo model for this study due to its robust performance and ease of access for prompting, fine-tuning, and testing via the OpenAI API. This accessibility ensures that even individuals with minimal experience can utilize the model effectively.

To address the first research question, we employed various prompting techniques, details of which are elaborated in subsequent sections. For the second research question, we tested the GPT-3.5 Turbo model by prompting the model using TSP instances with variant station numbers.

Lastly, to respond to the third research question, we set the model's temperature equal to 0.7 and prompted it multiple times with the same instance to assess performance enhancements achieved through self-ensemble methods.

### Dataset generation

In this research, we used a dataset of routes, where each route is a number of unique 2-dimensional points. To generate this dataset, we collected routes from the TSP-specific dataset TSPLIB, and we generated a set of random routes. We used routes from TSPLIB for in-context learning; meanwhile, the randomly generated routes were used for testing fine-tuning models.

We selected the TSPLIB 95 dataset as the base for our routes points dataset. TSPLIB is a library of sample instances for the TSP (and related problems) from various sources and types. Each file consists of a specification part and a data part. The specification part contains information on the file format and its contents. The data part contains explicit data. The route data can be stored in various formats. We selected files with points formatted in 2-dimensional coordinates, presenting the points in the training instances. The number of points in TSPLIB routes with the 2D point data type is variable and large. We collected 400 instances by taking the first 10 points of 400 routes’ files.

On the other hand, the testing dataset is generated randomly with a different schema. Because we want to test LLM response against routes with different numbers of stations, we created the data as follows:

- We selected this route size {10, 11, 12, 13, 14, 15, 16, 17, 18, 19, 20, 21, 22,}
- For each route size, we generate 30 different routes that have several points equal to the route size.

### In-context learning prompt engineering

**Fig 3:**
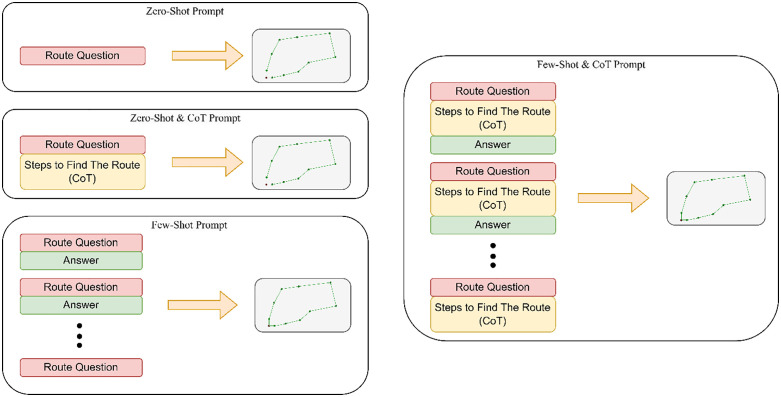
Different prompt structures. Zero-shot prompt general structure. Zero-shot Prompt structure with Chain of Thoughts part. Few-shot prompt. Few Shot prompts with the Chain of Thoughts part.

Prompt engineering is an intensive task with many trials and errors. For this task, we decided to prompt GPT3.5-turbo-0125. As suggested in [15], we select the model temperature to 0.7. We used many techniques to develop well-designed prompts and followed the recommended prompt-designing strategy to build our prompts. In this task, we use our generated testing data. As shown in Fig 3, we used four prompt formats, each applying a different combination of prompting techniques: zero-shot, zero-shot with CoT, few-shot, and few-shot with CoT. Each prompt contains context, model task, output format, and input data parts. In zero-shot prompts, we start with a context part that explains the needed knowledge to clarify the task and the formula used to calculate the distance between stations. The second part explains the prompt task. In the third part, we explained the expected format of the model output, and lastly, we added the input data of the stations. For zero-shot with CoT, we add a paragraph that explains how to do the task after the task explanation part. For both the few-shot and the few-shot with CoT prompts, we create them by adding 5 solved zero-shot or zero-shot with CoT examples of prompts and answers before the prompt we need to solve.

### Check model response feasibility

**Fig 4:**
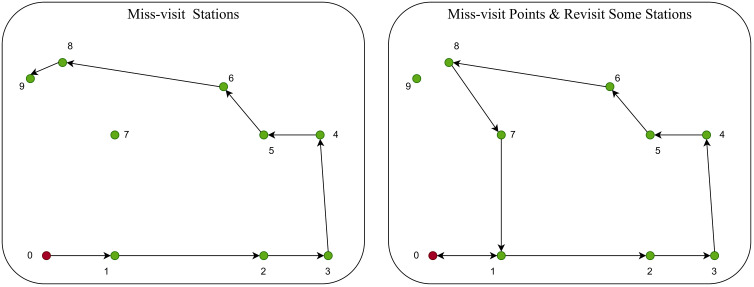
Model Hallucinates by doing Multiple-visits to Same Stations or Miss-visit Stations.

**Fig 5:**
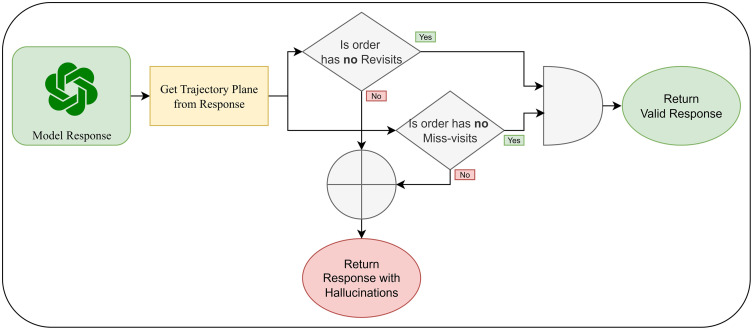
Hallucinations Checking Algorithm for Model Responses.

In the context of trajectory planning with LLMs, “hallucination” refers to instances where the model outputs routes that violate the Hamiltonian path condition [35], which requires visiting each city exactly once and returning to the starting point. A typical hallucination observed in our study involves the model revisiting certain nodes, miss-visiting some stations, or both, as shown in Fig 4. This type of error has been the most common hallucination encountered, guiding us to adhere to the recommended temperature for self-ensemble as suggested by [15] in their study on enhancing reasoning in language models through self-consistency (i.e., self-ensemble). To check hallucination, we follow the algorithm shown in Fig 5.

### Check model response quality

**Fig 6:**
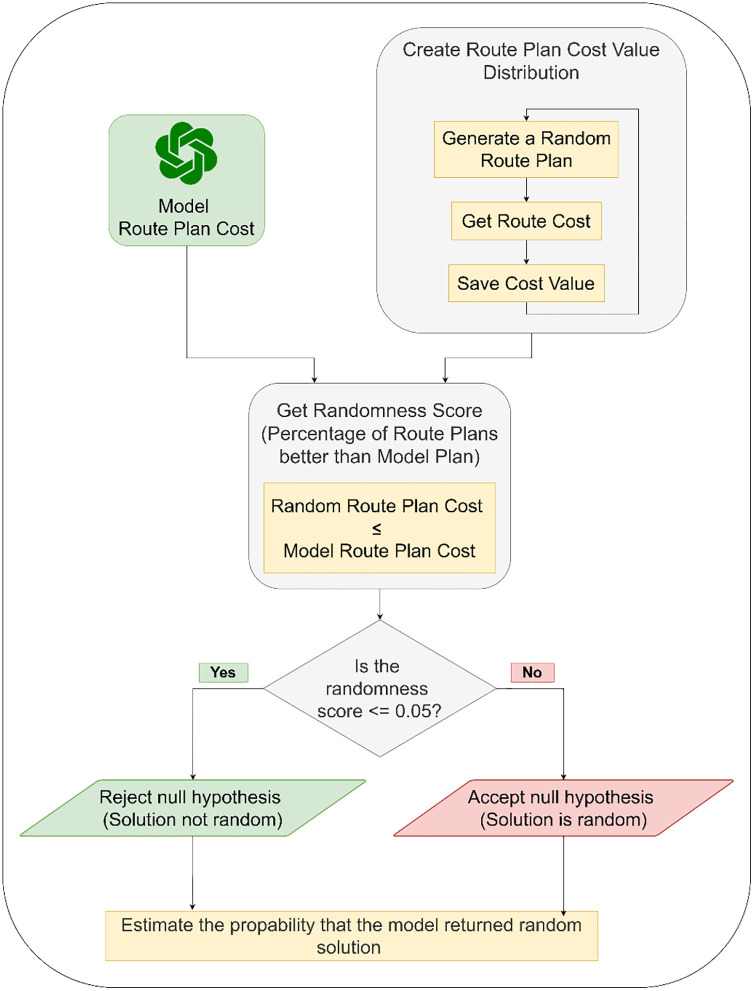
Null-hypothesis Testing Process.

In this research, we evaluated the created route using two distinct measures. The first measure, which we refer to as the randomness score, ranges from zero to one. As shown in Fig 6, to calculate this score, we created a normal distribution of routes total distances. We first randomly reorder the points in an instance—effectively generating a random route—and then evaluate the distance of these random routes. By repeating this randomization process and distance calculation, we create a normal distribution of distances for randomly generated routes. We then compare the cost of the model's routes to this distribution. Specifically, in (2), we count the number of random routes that have a distance less than or equal to that of the model's solution and divide this number by the total number of random solutions generated. This score allows us to test the null hypothesis for our model responses, clarifying the model’s ability to generate high-quality route plans. The randomness score effectively acts as the p-value for the solution being generated randomly. If the score is less than 0.05, we reject the null hypothesis and conclude that the route is not randomly generated. Conversely, a score higher than this threshold indicates that the model's route might be random. By repeating this procedure across multiple planned routes, we can estimate the likelihood that the model returns random solutions.

The second measure, known as the gap (3), calculates the difference between the distance of the route provided by the model and the optimal distance, normalized by the optimal distance. This widely recognized metric helps assess the efficiency of the proposed solution relative to the best possible outcome.


$$C_{R-valid}\;\leftarrow \;\{C_{R}\;\in \;Arr\_C_{R}\;|\;C_{R}\leq {\;C}_{M}\}$$ (1)

$$P\;\leftarrow \;\frac{C_{R-valid}\;}{N}$$ (2)

$$G\;\leftarrow \;\frac{C_{M}\;-\;C_{O}}{C_{O}}$$ (3)

Where:$C_{M}$: Model order cost

- $Arr\_C_{R}$: Random orders cost array
- $C_{R-valid}$: Random costs less than model costs
- N: Number of random orders
- P: Randomness score
- $C_{O}$: Optimal order cost
- G: Gap value

To implement self-ensemble in this study, we prompted the model eleven times with a temperature of 0.7 to obtain multiple responses for each route in the test dataset. We then assessed the effect of self-ensemble using varying ensemble sizes: {1,3,5,7,9,11}. For instance, to determine a solution for an ensemble of size B, we would select the first B responses from the eleven obtained. Next, we filtered out any responses that were deemed to be hallucinations, we calculated the cost of each solution, and selected the one with the lowest cost as the definitive solution for that particular instance, as shown in (4, 5, 6, 7, 8).


B ∈ {1,3,5,7,9,11}; (4)

$${\text{Response}\_\text{Arr}}_{\text{B}}\;\leftarrow \{{\text{r}}_{\text{i}}|{\text{r}}_{\text{i}}\in \text{ Respons}\text{e}\_\text{Arr},\text{i}=\text{1},\ldots ,\text{B}\}$$ (5)

$${Response\_Arr}_{valid}\;\leftarrow \;\left\{r\;\in \;{Response\_Arr}_{B}\right|\neg isHallucination(r)\}$$ (6)

$$r^{*}\;\leftarrow {argmin}_{\text{r }\in \;{Response\_Arr}_{valid}}\text{ C}(\text{r})$$ (7)

$${\text{Response}\_\text{Arr}}_{\text{best}}\;\leftarrow \;\left\{\text{r }\in \;{\text{Respons}{\text{e}}_{\text{Arr}}}_{𝐛}\right|\text{r}={\text{r}}^{*}\},\text{ C}({\text{r}}^{*})$$ (8)

Where:

- Response_Arr: The set of all responses for a particular route in the test dataset
- B: Ensemble size
- r*: The best response
- C(r): The cost function that assigns a cost to each response r
- isHallucination(r): is a predicate function that evaluates to true if the response r is considered a hallucination

### Heuristic solution (evolution algorithm)

**Fig 7:**
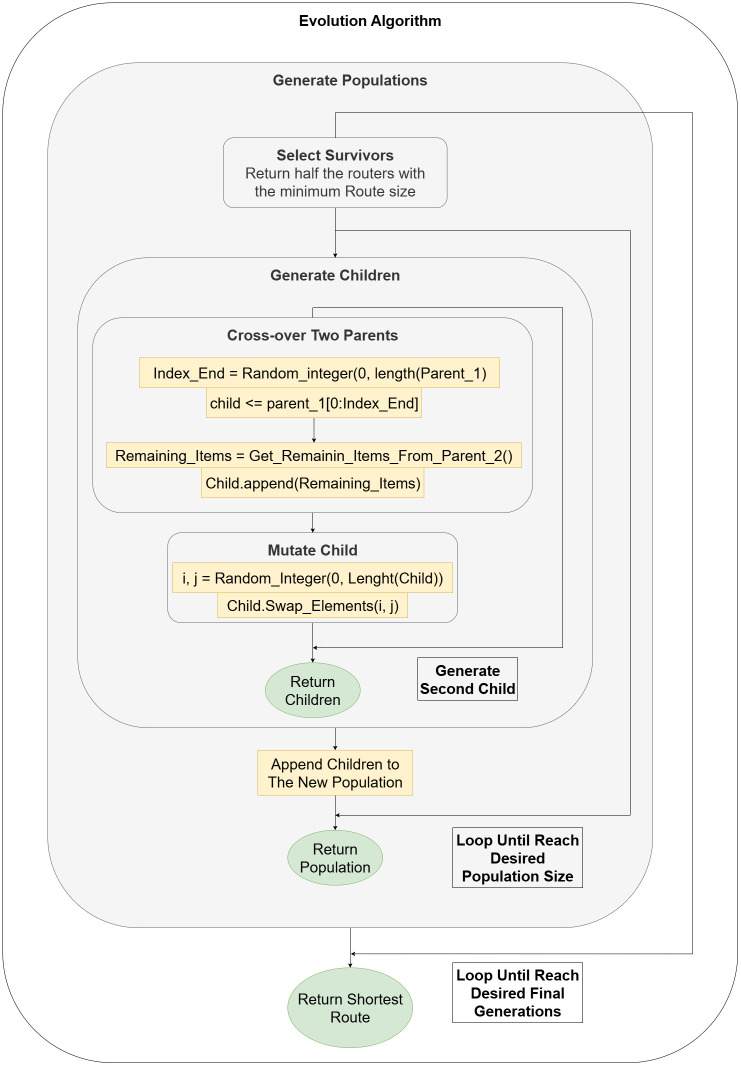
Evolution algorithm diagram.

We implemented a heuristic algorithm (evolution algorithm) to compare our model performance against a heuristic-based technique. As shown in Fig 7, we start with a population of randomly generated routes, then we iterate 10 times to generate new generations. In each generation, we select half of the previous generation and append the population with the generated children by doing cross-over between parents, and apply mutation to each child. At last, we get the route with the minimum route distance from the final population.

### Data normalization

**Fig 8:**
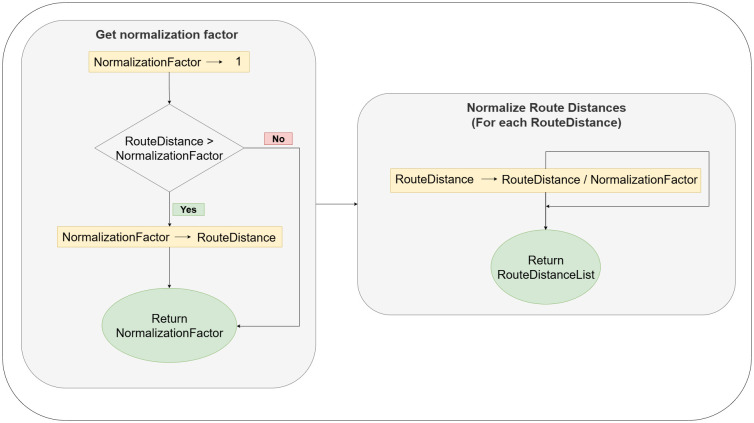
The Routes distance normalization process.

In order to compare the total distance of generated routes, we normalize the route’s distance to a value between 0 and 1. As shown in Fig 8, we normalize the route distances to show the difference between various prompting and optimization techniques. First, we loop over route distances to get the biggest route distance to get the normalization factor. Then, we devise a route distance by the normalization factor to conserve the variations between all generated routes.

### Experiment results

In this section, we will discuss the outcomes of our experiments. Initially, we analyze the percentage of correct trips returned by the in-context learning techniques, without considering the quality of these trips. A correct trip must follow the Hamiltonian path condition. It should begin and end at the same location and visit each station exactly once. Subsequently, we step into the quality of these correct trips for the in-context learning scenarios. We further examine how self-ensemble techniques enhance the proportion of correct trips returned for each instance size and boost the overall quality of the routes.

### In-context learning techniques effect on route planning

**Fig 9:**
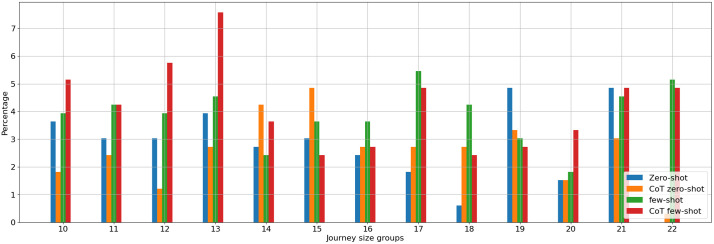
Good Order Percentage for Multiple Groups of Similar route Size and Different Prompting Techniques.

In Fig 9, the bar chart illustrates the results from analyzing 30 TSP instances across sizes ranging from 10 to 22, highlighting the correlation between instance size and the percentage of correct, non-hallucinated responses from a language model employing various in-context learning strategies, such as zero-shot and few-shot learning, both with and without the embedding of CoT technique. Meanwhile, the overall success rates are less than 10 percent, which displays remarkable differences between the in-context learning approaches. Few-shot and few-shot with CoT techniques display promising accuracy for smaller instances, particularly those close to the size of 10. Consequently, the few-shot approach appears to uphold precision more consistently, even as instance sizes grow, underlining the benefit of providing the model with a set of examples, assuming the constraints of maximum token capacity are met. These findings show the significance of prompt engineering, which proves important not only for generating accurate responses but also for adhering to the model's token limitations.

### Self-ensemble effect on route planning

**Fig 10:**
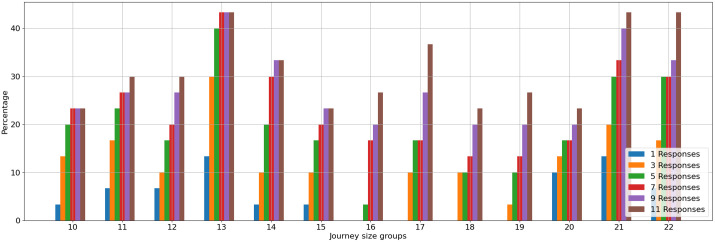
Good Order Percentage for Multiple Groups of Similar Route Size, Applying Self-Ensemble on Few-Shot with CoT Prompts Responses.

In Fig 10, the bar graph depicts the model's performance. Whereas the self-ensemble effect is independent of the in-context learning technique, we decided to show the results for a few shots with CoT prompts. Each instance has 11 self-ensemble responses. The data show the self-ensemble's positive impact, enhancing the accuracy of correct trips. The model reliably produced at least one accurate, non-hallucinated trip for smaller instances, with the self-ensemble's advantage becoming more distinct as instance size grew; for example, for size 22, a bigger ensemble number improved the rate of correct, non-hallucinated solutions.

### In-context trip quality analysis

**Fig 11:**
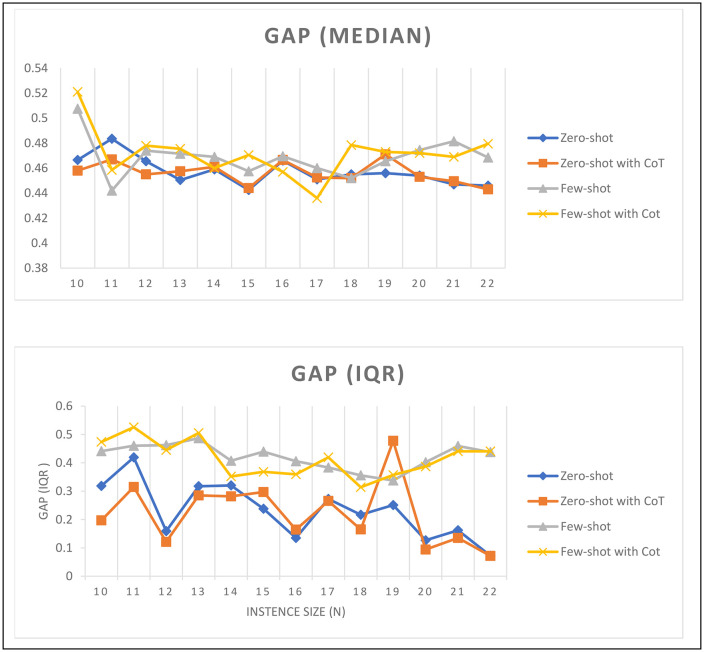
Median and interquartile for the gap of each group of routes with similar size and different in-context prompting technique for.

Fig 11 depicts the gap median and interquartile range IQR values across various in-context learning techniques, offering a clear visualization of the model’s performance as the instance size increases. This analysis clarifies the ability of LLMs to generate valuable route plans. For zero-shot and zero-shot with CoT, we found consistent median values regardless of the instance size increase. However, few-shot and few-shot with CoT show a consistence upward trend in the median gap. This trend suggests that the challenges associated with solving drone routing grow as the problem size grows. Interestingly, incorporating CoT does not appear to significantly alter this trajectory, indicating that the complexity of larger instances presents a uniform challenge across different techniques. For IQR, we observe a declining trend for zero-shot, and zero-shot with CoT; however, few-shot and few-shot with CoT show a more consistence value with instance size increase. The fluctuation in the IQR suggests variability in the model's performance consistency. It is evident that while the median performance deteriorates as the instances grow, the variation in performance measured by the IQR does not align with a straightforward trend, indicating that certain instance sizes may present unique challenges or advantages that are not solely dependent on the size of the problem.

Table 1 provides randomness scores for solutions to the model-planned routes spanning various instance sizes. These scores compare zero-shot and few-shot in-context learning techniques, with and without the integration of CoT. To evaluate the randomness of the solutions, the median scores and their IQR are analyzed. The median scores reveal that zero-shot approaches frequently result in higher randomness scores, often surpassing the 0.05 threshold. This suggests that the solutions might be random, and we cannot reject the Null hypothesis, which posits that the model generates random solutions (i.e., low-quality route plans). Contrary to expectations, the addition of CoT to zero-shot learning did not significantly decrease the randomness scores. In contrast, few-shot methods generally produce lower median randomness scores compared to zero-shot, indicating a tendency toward less random solutions. Similarly, the incorporation of CoT with few-shot learning did not lead to improved randomness scores.

**Table 1:** Interquartile and Median for P-values of each group of routes with similar size and different in-context prompting techniques.

		10	11	12	13	14	15	16	17	18	19	20	21	22
**Zero-shot**	IQR	0.32	0.42	0.16	0.32	0.32	0.24	0.14	0.27	0.22	0.25	0.13	0.16	0.07
	Median	0.47	0.48	0.47	0.45	0.46	0.44	0.47	0.45	0.46	0.46	0.45	0.45	0.45
**Zero-shot & CoT**	IQR	0.20	0.31	0.12	0.29	0.28	0.30	0.16	0.27	0.17	0.48	0.09	0.14	0.07
	Median	0.46	0.47	0.46	0.46	0.46	0.44	0.47	0.45	0.45	0.47	0.45	0.45	0.44
**Few-shot**	IQR	0.44	0.46	0.46	0.49	0.41	0.44	0.41	0.38	0.36	0.34	0.40	0.46	0.44
	Median	0.51	0.44	0.47	0.47	0.47	0.46	0.47	0.46	0.45	0.47	0.47	0.48	0.47
**Few-shot & CoT**	IQR	0.47	0.53	0.45	0.51	0.35	0.37	0.36	0.42	0.31	0.36	0.39	0.44	0.44
	Median	0.52	0.46	0.48	0.48	0.46	0.47	0.46	0.44	0.48	0.47	0.47	0.47	0.48

**Fig 12:**
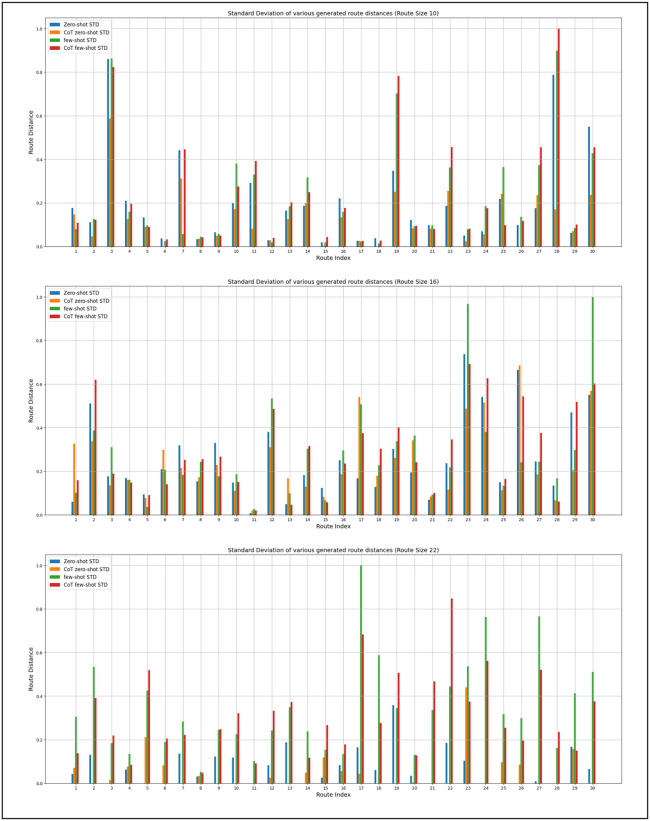
Standard Deviation for various journey sizes.

In Fig 12, we demonstrate the variation between the standard deviation for various promoting techniques. Each graph represents 30 different routes with the same size for sizes 10, 16, and 22. The standard deviation is calculated for 11 different route generations for the same journey.

We found that the CoT with the Zero-Shot technique has the smallest standard deviation in small-size journeys; meanwhile, few-shot techniques with and without CoT show the largest values of standard deviation. In medium-sized journeys, we observed that most journeys have similar standard deviation values, although a few journeys have spikes in value. In medium-sized journeys, Zero-Shot with CoT still has an advantage over other techniques; however, Zero-Shot techniques started to show degradation in standard deviation values. In larger journeys, we found that the zero-shot technique shows the lowest values, indicating the least variation between values. As zero-shot has the smallest prompt size. This leap in performance might be due to the increased prompt size for larger journeys, which have the least impact on zero-shot prompts.

### Improving TSP solution quality with self-ensemble

**Fig 13:**
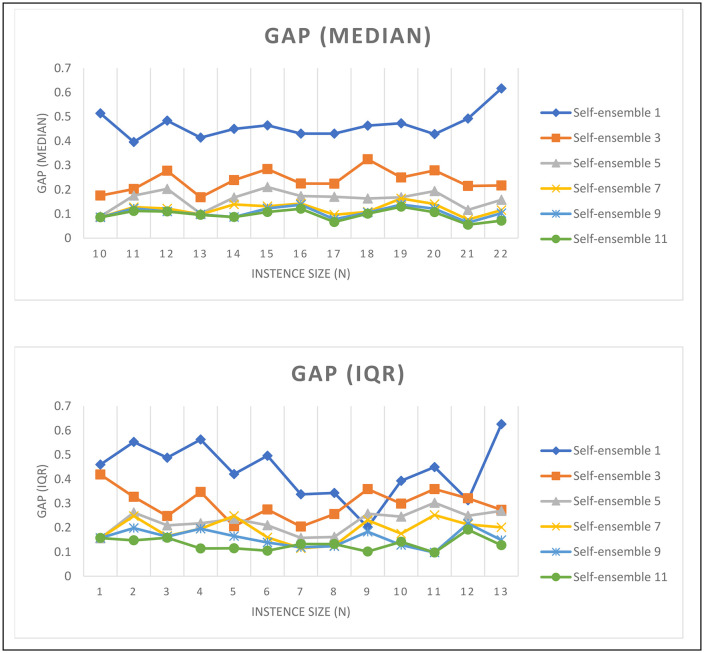
Median and interquartile for the gap of each group of routes with similar size and different self-ensemble values for fine-tuned model.

In Fig 13, the graphs display gap statistics (i.e., Median and IQR) for route planning, showcasing the quality of the valid routes returned out of 30 instances evaluated at each size. Despite being fine-tuned on size-10 instances only, the model exhibits good generalization to larger sizes. However, an increasing trend is noticeable as instance sizes increase beyond 10, indicated by rising gap median and interquartile range (IQR) values. Notably, implementing self-ensemble techniques shows a marked improvement in solution quality. This is evidenced by reduced median gap and IQR across all instance sizes. Moreover, the enhancement in trip quality is proportional to the ensemble size within the explored range, highlighting the benefit of self-ensemble in optimizing the performance of LLM response for combinatorial tasks like the TSP. The above analysis provides a detailed insight into the capabilities of the model in finding efficient routes. As the size of the problem increases, both the median of the gap and the Interquartile Range (IQR) statistics tend to increase, yet the randomness scores suggest that the solutions are not by chance. This is particularly true for larger problems, where the large number of possible solutions makes the probability of generating a good one by chance very low. Even when there is a large gap statistic, most solutions can pass the randomness test, indicating that the model is effectively solving problems rather than randomly selecting the order of stations.

Table 2 shows randomness scores for solutions to the self-ensemble routes spanning various instance sizes. These scores compare zero-shot and few-shot in-context learning techniques, with and without the integration of CoT. To evaluate the randomness of the solutions, the median scores and their IQR are analyzed. Meanwhile, these values for zero-shot show promising results; the few-shot impact on the result reduced the median to obvious values. Also, CoT embedding in the prompt has an effective impact on the quality of the results. Large number of possible solutions makes the probability of generating a good one by chance very low. Even when there is a large gap statistic, most solutions can pass the randomness test, indicating that the model is effectively solving problems rather than randomly selecting the order of nodes.

**Table 2:** Interquartile and Median for the P-values for each group of routes with similar size.

		10	11	12	13	14	15	16	17	18	19	20	21	22
Self-ensemble 1	**IQR**	0.04	0.09	0.04	0.15	0.14	0.13	0.24	0.30	0.16	0.31	0.43	0.70	0.95
	**Median**	0.02	0.04	0.03	0.07	0.04	0.05	0.13	0.30	0.20	0.20	0.32	0.39	0.50
Self-ensemble 3	**IQR**	0.02	0.04	0.03	0.05	0.05	0.06	0.07	0.14	0.14	0.12	0.21	0.29	0.14
	**Median**	0.02	0.02	0.02	0.02	0.04	0.03	0.06	0.08	0.12	0.08	0.18	0.18	0.20
Self-ensemble 5	**IQR**	0.02	0.03	0.02	0.03	0.04	0.05	0.07	0.10	0.08	0.09	0.17	0.16	0.12
	**Median**	0.01	0.02	0.01	0.02	0.02	0.03	0.06	0.06	0.07	0.06	0.15	0.13	0.16
Self-ensemble 7	**IQR**	0.02	0.02	0.02	0.03	0.04	0.03	0.06	0.11	0.08	0.07	0.13	0.11	0.10
	**Median**	0.01	0.02	0.01	0.02	0.02	0.02	0.03	0.06	0.07	0.06	0.13	0.11	0.14
Self-ensemble 9	**IQR**	0.02	0.02	0.03	0.03	0.02	0.03	0.06	0.08	0.08	0.08	0.12	0.07	0.13
	**Median**	0.01	0.01	0.01	0.02	0.02	0.02	0.02	0.06	0.06	0.06	0.09	0.09	0.13
Self-ensemble 11	**IQR**	0.02	0.02	0.03	0.02	0.02	0.03	0.06	0.06	0.08	0.07	0.12	0.06	0.13
	**Median**	0.01	0.01	0.01	0.02	0.01	0.02	0.02	0.05	0.06	0.06	0.09	0.08	0.12

### Compare generated routes to various standard techniques

**Fig 14:**
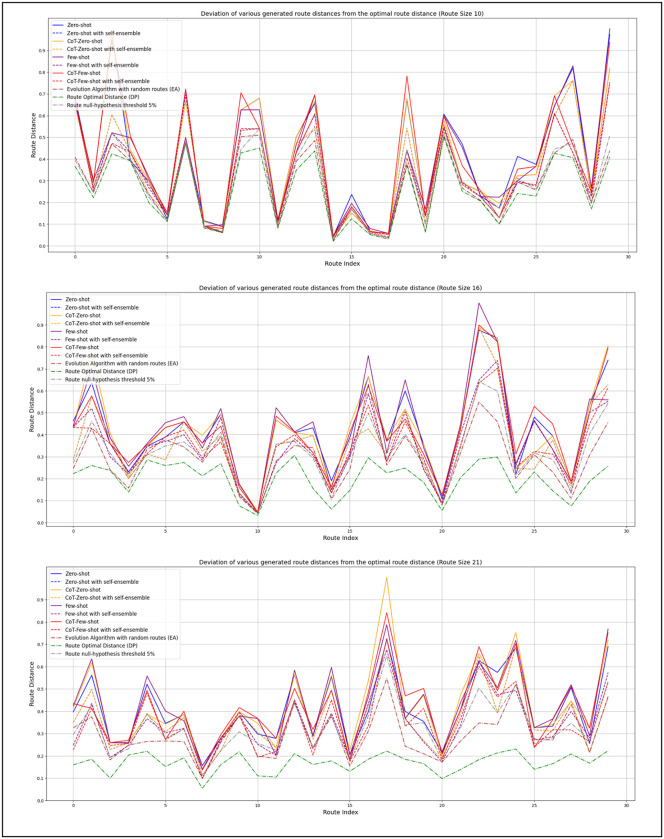
Comparing generated routes to various standard techniques.

Fig 14 illustrates the deviation of route distances generated by various prompting techniques, both with and without the application of self-ensemble methods. In the self-ensemble approach, the model is prompted 11 times with identical input (i.e., the same set of delivery points), and the most optimal route among the outputs is selected. All route distances are normalized to a range between 0 and 1 to enable meaningful comparison across different instance sizes. Each plotted route corresponds to a fixed number of nodes but varies in the path chosen. These normalized distances are compared to the corresponding optimal route distances, and heuristic-based routes created by a heuristic-based algorithm (evolution algorithm), and evaluated against a 5% p-value threshold derived from a null hypothesis test. The threshold represents whether a given route falls within the shortest 5% of a distribution constructed from 10,000 randomly generated routes for the same instance, thus serving as a statistical indicator of solution quality.

To capture the effect of increasing instance complexity, three subfigures represent journeys consisting of 10, 16, and 21 delivery points, respectively. For small-scale instances (e.g., 10 nodes), most prompting strategies—regardless of the presence of self-ensemble—demonstrate relatively similar performance. In the majority of cases, deviations from the optimal route remain under 0.05. Notably, the Few-Shot with CoT technique combined with self-ensemble achieves the smallest deviations, with many of its solutions falling below the null-hypothesis threshold, indicating statistically significant performance.

As the instance size increases to 16 nodes, we observe a broader range of performance across different prompting strategies. Although the overall deviation from optimal solutions increases, several techniques still produce route distances below the randomness threshold, showing robustness in moderately complex scenarios. For the largest tested instances (21 nodes), deviations become more pronounced across all prompting methods, reflecting the increased difficulty of the routing task. However, a number of routes—particularly those generated with self-ensemble—continue to reject the null hypothesis, affirming that the LLM is capable of producing high-quality outputs, especially with self-ensemble involved. Interestingly, the Few-Shot performs least favorably in this setting. This degradation may be attributed to poor description of the task or the increased prompt length approaching the model's context window limit, which can impair the model’s reasoning capabilities due to token constraints.

We compare our model results to routes generated by applying a heuristic-based approach (evolution algorithm) applied to randomly order routes. From our observation, the heuristic route's performance is almost similar to our model performance for small and medium routes, e.g., (10 nodes, 16 nodes). However, our model’s close efficiency to the heuristic approach for larger routes, such as 21 nodes, the heuristic approach shows better performance in most cases.

## Conclusion and future work

This research explores the potential of using LLMs to solve the TSP combinatorial problem in the context of the drone routing problem (a combinatorial optimization task critical to efficient drone-based delivery systems). Utilizing OpenAI’s GPT-3.5 Turbo model, we focused on analyzing the performance of various prompting strategies—including zero-shot, few-shot, and CoT techniques—across multiple problem sizes. We have uncovered several key insights. Also, the implementation of self-ensemble methods has further enhanced the model's performance, delivering improved solution quality.

Our results confirm that few-shot and few-shot with CoT demonstrate increased accuracy and structure of model-generated routes for smaller instances. As problem size increases, these strategies maintain relatively better performance compared to zero-shot methods, though the quality of solutions degrades with complexity. This underlines the benefit of providing the model with a set of examples, considering that the constraints of maximum token capacity are met. The integration of self-ensemble techniques further enhances performance, especially in larger problem instances, by increasing the likelihood of generating at least one valid and high-quality solution. Our model showed a comparable performance to a heuristic-based technique, which shows the model's ability to generate efficient routes.

The maximum token limit imposed on both prompt and model response was found to have a direct impact on the model performance. As problem instance size increases, models exhibit increased hesitations and a higher incidence of incomplete responses. This scalability limitation can be addressed by selecting models with a token window size that is appropriately matched to the expected complexity and length of the target problem instances.

Moreover, the gap analysis and randomness testing reveal that, even when the solutions are not optimal, they are often non-random and reflect meaningful reasoning. Few-shot approaches consistently produced lower randomness scores, indicating more deliberate route planning by the model. The effectiveness of self-ensembling in reducing both gap statistics and performance variability underscores its importance as a robust enhancement for LLM-driven optimization.

In conclusion, this study highlights the feasibility and limitations of employing in-context learning with LLMs for drone routing. While challenges remain—particularly in scaling to larger instances under token constraints—the findings support continued exploration of LLMs as adaptive, data-efficient optimizers. Future research should investigate hybrid methods that combine LLM reasoning with traditional optimization post-processing, as well as fine-tuning strategies to improve generalization and scalability.

## Supporting information

S1 File Data available at the attached Link. (DOCX)
