# Supplementary material for: Employing in-context learning prompts with large language models for drone routing in delivery services
Source: PLoS One. 2026 Mar 27;21(3):e0321917. doi: 10.1371/journal.pone.0321917 (PMC13028478; doi:10.1371/journal.pone.0321917)
Supplement: S1 File — (DOCX) [file pone.0321917.s001.docx]

Data available at the following Link :

[Solve-TSP-using-GPT3.5/generated_data at main · ahmed-abdulhuy/Solve-TSP-using-GPT3.5 · GitHub](https://github.com/ahmed-abdulhuy/Solve-TSP-using-GPT3.5/tree/main/generated_data)
